# Adaptation and performance evaluation of Perendale, Dorper, and Suffolksheep in Bangladesh

**DOI:** 10.5455/javar.2025.l986

**Published:** 2025-12-25

**Authors:** Nure Hasni Desha, Sadia Afrin, Md. Mahmudul Hasan Pasha, Md Nahid Hassan Chawdhury, Md. Zillur Rahman

**Affiliations:** 1 Bangladesh Livestock Research Institute Regional Station, Rajshahi, Bangladesh; 2 Sheep Production Research Division, Bangladesh Livestock Research Institute, Dhaka, Bangladesh

**Keywords:** Dorper sheep, Exotic sheep, Perendale sheep, Performance evaluation, Suffolk sheep

## Abstract

**Objective:**

The research was conducted to adapt the Perendale, Dorper, and Suffolk sheep and evaluate their productive and reproductive performances.

**Materials and Methods:**

Data from 82 pure exotic sheep were recorded within the period of 2016–2024. The considered traits were birth weight, live weight at 3 (BWT3), 6 (BWT6), 9 (BWT9), and 12 months (BWT12), adult weight (2–3 years), average daily weight gain at 0–3 (ADG0-3), 3–6 (ADG3–6), 6–9 (ADG6–9), and 9–12 (ADG9–12) months of age, wool production, age at first conception, gestation length, litter size, age at first lambing, lambing interval, and days open. The data were analyzed using R software version 4.4.2.

**Results:**

Breed had no significant effect on productive and reproductive traits except BWT3, ADG0-3, wool production per sheep per shearing, and average lambing interval. The highest BWT3 (kg) and ADG0-3 (gm/day) were found in Dorper sheep, followed by Suffolk and Perendale, and the values were 23.15, 22.19, and 19.39 and 216.52, 210.86, and 173.12, respectively. In the case of wool production per sheep per shearing (kg), the highest production was found in Suffolk compared to Perendale and Dorper, respectively. The lowest lambing interval (days) was found in Dorper sheep, followed by Suffolk and Perendale, respectively. The average survivability rate of lambs and growing sheep was 96.34 and 98.73, respectively.

**Conclusion:**

The findings indicate that exotic sheep are well-adapted to our environment, and Dorper sheep may be utilized to produce meat-type crossbred sheep.

## Introduction

Bangladesh is a country with a diverse range of livestock species, where small ruminants play a significant role, and sheep constitute the third-largest livestock population, numbering 3.903 million [1]. The sheep enterprises in Bangladesh have historically relied on indigenous types, contributing significantly to the improvement of the livelihoods of landless, marginal, and small-scale farmers, as well as to the country's income generation [2–8]. Indigenous sheep are well-adapted to the local environment, with minimal feed requirements, higher production performance, greater disease resistance, and superior meat quality [9-11]. However, they often exhibit lower growth rates and wool quality compared to foreign breeds [12,13].

Several programs have been implemented to introduce exotic sheep germplasm with the aim of improving native sheep in the country, but these efforts have not been sustained in the long run [14]. In 2016, the Bangladesh Livestock Research Institute (BLRI) imported 42 sheep of three exotic breeds (Suffolk, 13; Perendale, 14; and Dorper, 15) from Australia to enhance the production performance of native sheep [15]. These breeds were chosen for their well-established productive and reproductive attributes, which are widely recognized in terms of growth performance, fertility, and meat quality. The primary goal of introducing these breeds at BLRI was to assess their adaptability and improve the productivity of local sheep populations through crossbreeding programs. Furthermore, selective breeding among these imported breeds has been undertaken to proliferate their numbers and assess their suitability for scaling up. Suffolk is a British breed known for its fast growth, high-quality meat, and ease of lambing [16].

Perendale, established in New Zealand, is known for its dual-purpose meat and wool production, easy-care lambing, good mothering, and lamb survival, as well as its ability to flourish in harsh, hilly terrain [17]. Dorper, which originated in South Africa, is recognized for its hardiness, rapid weight increase, and adaptability to a wide range of climatic conditions, including desert settings [18-21]. Each of these breeds has been extensively studied in its country of origin, where it has been optimized through decades of selective breeding programs. However, their performance in tropical environments, such as Bangladesh, characterized by high environmental temperatures, high humidity, seasonal flooding, and a prevalence of diseases, remains largely undocumented. To make informed decisions about the potential of these breeds, it is essential to have a thorough understanding of their overall performance, including sexual maturity, prolificacy, lambing interval, milk production, body weight gain, disease susceptibility, and mortality rates, in our country. This study will show a detailed comparison between the productive and reproductive performance of these imported sheep breeds in Bangladesh and the established benchmarks from their countries of origin. By evaluating their performance, this research will help to understand whether these foreign breeds can be sustainably utilized to enhance sheep production in Bangladesh, as well as their effective integration into the national livestock development strategy. Thus, the research aimed to evaluate the productive, reproductive, and adaptation performance of Perendale, Dorper, and Suffolk sheep in Bangladesh.

## Materials and Methods

### Ethical approval

The Animal Experimentation and Ethics Committee of Bangladesh Livestock Research Institute approved this study project (Reference no.: AEEC/BLRI00117/2023). All rules and regulations for animal care were strictly adhered to during animal handling.

### Location of the study area

The experiment was conducted at the BLRI Exotic Sheep Research Farm in Savar, Dhaka. The average daily temperature and relative humidity of the area range from 15°C to 34°C and 48.71%–85.9%, respectively.

## Flock management

### Experimental animal

**Figure 1.**
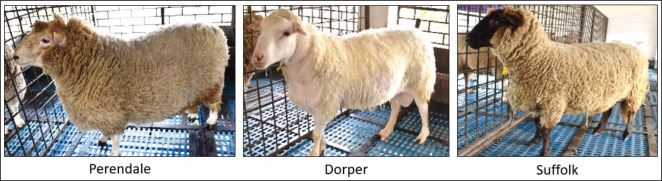
Different breeds of sheep.

Data from a total of 82 pure exotic sheep (Dorper, Perendale, and Suffolk) were managed from 2016 to 2024. The experimental population comprised 25 males and 29 females of the Perendale breed, 9 males and 6 females of the Dorper breed, and 5 males and 8 females of the Suffolk breed, respectively (Fig. 1).

### Housing and feeding

Animals were kept in intensive management in a permanent slatted-floor house elevated above the ground level. At 1.5% of their body weight, the animals were fed a concentrate diet in the morning that included 17% CP and 11 MJ ME/kg DM, which was based on broken maize, wheat bran, khesari bran, soybean meal, and protein concentrate, as well as green grasses (Napier pakchong, Oats, Maize, and Australian sweet jumbo), which were provided on an *ad libitum* basis. Fresh water was supplied in adequate quantities at all times. The concentrate mixture was formulated using the following feed ingredients: wheat bran (30%), broken maize (30%), soybean meal (20%), khesari bran (16%), protein concentrate (1.5%), di-calcium phosphate (1%), salt (1%), and vitamin–mineral premix (0.5%).

### Breeding

To preserve the pure bloodline, the breeding program was managed in a way that minimized inbreeding. An estrous female naturally mated with the ram in accordance with the established mating chart. Throughout the year, ewes were permitted to produce lambs as part of a natural, controlled breeding scheme.

### Health and disease

Sheep were vaccinated against peste des petits ruminants and treated regularly to manage both internal and external parasites through deworming and dipping with a 0.5% malathion solution. To maintain biosecurity, a footbath containing potassium permanganate solution was always managed in front of the animal shed.

### Shearing

Wool was sheared twice a year in March–April and September–October.

### Traits studied

The productive traits considered were birth weight (kg), live weight (kg) at 3 (BWT3), 6 (BWT6), 9 (BWT9), and 12 (BWT12) months, adult weight (2–3 years, kg), average daily weight gain (gm/day) at 0–3 (ADG0–3), 3–6 (ADG3–6), 6–9 (ADG6–9), and 9–12 (ADG9–12) months of age, and wool production (per sheep per shearing, kg). The reproductive traits considered were age at first conception (in days), gestation length (in days), litter size (number of lambs), age at first lambing (in days), lambing interval (in days), and days open (in days). In addition, the prevalence of disease and the survivability of lambs were also documented. The average daily gain was calculated by the following formula:

ADG (gm/day) = {(final live weight (kg)—initial body weight (kg))/number of days} × 1,000

The results obtained were compared with the published performance records of the breeds (Table 1).

**Table 1.** Productive and reproductive performance of different exotic sheep.

Parameters	Breed			References
	Perendale	Dorper	Suffolk	
Birth weight (kg)		3.33		[22]
		4.09		[23]
		4.12		[24]
		5.5	5.7	[25]
			4.94	[26]
			4.8	[27]
	4.21			[28]
	4.30			[28]
3-month body weight (kg)		23.8		[23]
			25.57	[26]
	21.5			[28]
	23.8			[28]
6-month body weight (kg)		36.7		[24]
			48.7	[29]
9-month body weight (kg)		47.8		[24]
12-month bodyweight (kg)		63.5		[24]
	34.6			[28]
	40.4			[28]
ADG_0-3_ (gm/day)		240-280		[30]
		219.0		[23]
			300	[26]
ADG_3-6_ (gm/day)		180		[30]
Gestation length (day)		147		[30]
			146.20	[26]
Litter size (number)		1.54		[30]
			1.63	[26]
			1.8	[27]
	1.75			[28]
	1.32			[28]
Age at first lambing (day)		570		[22]
			717.9	[29]
Average lambinginterval (day)		398		[22]
Survivability of lamb (%)		91.0		[30]
		93.4		[23]
		81.0		[25]

Breed, generation, and season were considered as the fixed effects. The year-round seasonal impacts were noted as early summer (March–April), late summer (May–June), rainy season (July–October), and winter (November–February).

### Experimental design and statistical analysis

The sample size was unbalanced because of natural service and breed differences. The experimental design was a 3-factor completely randomized design (CRD 3 factor). The model is used to determine the effect of breed, generation, and season on different productive and reproductive performances:

*Y_ijk_* = μ + *B_i_* + *G_j_* + *S_k_* + *e_ijk_*

Where,

*Y_ijk_* = Record of *Y^th^* animal born in *j^th^* breed under jthgeneration in *K^th^* season

μ = Overall population mean for any of the considered trait

*B_i_* = Effect of *i^th^* breed (where *i* = Perendale, Dorper and Suffolk sheep breed)

*G_j_* = Effect of *j^th^* generation (where *j* = first, second and third generation)

*S_k_* = Effect of *k^th^* season of birth (where *k* = early summer, late summer, rainy, and winter season)

*e_ijk_* = Random residual error

Recorded data were assembled into a Microsoft Excel worksheet, arranged, and analyzed by R-software version 4.4.2 with the “doebioresearch” package. The Shapiro-Wilk normality test was performed to measure the normality of the data. Mean comparison test was performed by Duncan’s Multiple Range Test. To compute the multiple bar graph and disease graph, the “ggplot2,” “patchwork,” and “ggstatsplot” packages were used.

## Results and Discussion

### Productive performance

The productive performance of exotic sheep is shown in Table 2. Breed had no significant effect on productive traits except BWT3, average ADG0-3, and wool production per sheep per shearing. The highest BWT3 (kg) and ADG0-3 (gm/day) were found in Dorper sheep, followed by Suffolk and Perendale, and the values were 23.15, 22.19, and 19.39; and 216.52, 210.86, and 173.12, respectively. In the case of wool production per sheep per shearing (kg), the highest production was 2.02, followed by 1.73 and 0.46, found in Suffolk, Perendale, and Dorper, respectively. There was no significant effect of season on any of the production traits, except for birth weight (Table 3). The highest birth weight was observed in the winter season, followed by early summer, the rainy season, and late summer, with values of 3.95, 3.72, 2.80, and 2.35 kg, respectively.

**Table 2.** Average productive and reproductive performance of exotic sheep.

Parameters	Breed			SEM	*p*-value
	Perendale	Suffolk	Dorper		
Production traits-					
Birth weight (kg)	3.79 (54)	3.22 (13)	3.67 (15)	0.1948	0.1923
BWT3 (kg)	19.39^b^ (52)	22.19^ab^ (13)	23.15^a^ (15)	1.0343	0.03139
BWT6 (kg)	26.99 (51)	29.09 (12)	32.11 (15)	1.5221	0.08321
BWT9 (kg)	32.61 (48)	34.97 (12)	37.85 (15)	1.8168	0.1459
BWT12 (kg)	39.94 (42)	40.88 (11)	45.28 (12)	2.1588	0.2754
Adult weight (kg)	61.21 (32)	57.20 (09)	67.73 (09)	3.6511	0.321
ADG_0-3_ (gm/day)	173.12^b^ (52)	210.86^a^ (13)	216.52^a^ (15)	10.76	0.009646
ADG_3-6_ (gm/day)	82.51 (51)	76.66 (12)	99.48 (15)	7.51	0.2367
ADG_6-9_ (gm/day)	60.12 (48)	71.48 (12)	63.78 (15)	5.5061	0.4387
ADG_9-12_ (gm/day)	63.36 (42)	56.87 (11)	70.46 (12)	6.2202	0.5329
Wool production/sheep/ shearing (kg)	1.73^a^ (04)	2.02^a^ (04)	0.46^b^ (04)	0.3139	0.01484
Reproduction traits-					
Age at first conception (day)	584.94 (16)	677.00 (04)	695.00 (03)	47.083	0.2599
Gestation length (day)	145.19 (16)	144.25 (04)	146.67 (03)	0.5702	0.1591
Litter size (number)	1.25 (16)	1.42 (04)	1.22 (03)	0.134	0.6885
Age at first lambing (day)	729.81 (16)	788.50 (04)	841.67 (03)	44.9156	0.3215
Average lambing interval (day)	635^b^ (08)	383^a^ (04)	382.5^a^ (02)	70.4999	0.03348
Days open (day)	152.63 (16)	186.25 (04)	149.67 (02)	26.0233	0.694

BWT3 = Body weight at 3 months of age; BWT6 = Body weight at 6 months of age; BWT9 = Body weight at 9 months of age; BWT12 = Body weight at 12 months of age; ADG = Average daily weight gain; SEM = Standard error of the mean; Figure in parentheses indicates the number of observations. ^a,b^ means with different superscripts differed significantly within the row (*p* < 0.05).

**Table 3.** Effect of season of birth on productive and reproductive traits.

Parameters	Season				SEM	*p*-value
	Early summer	Late summer	Winter	Rainy		
Production traits-						
Birth weight (kg)	3.72^a^ (46)	2.35^b^ (06)	3.95^a^ (28)	2.80^ab^ (02)	0.213	0.004594
BWT3 (kg)	20.44 (45)	18.12 (05)	20.84 (28)	25.20 (02)	1.188	0.52119
BWT6 (kg)	29.05 (44)	22.52 (05)	27.51 (28)	37.40 (02)	1.744	0.14815
BWT9 (kg)	35.34 (40)	27.00 (05)	32.62 (28)	45.35 (02)	2.036	0.06939
BWT12 (kg)	41.72 (38)	30.70 (05)	41.45 (20)	51.35 (02)	2.441	0.08468
Adult weight (kg)	61.40 (28)	35.00 (01)	61.67 (19)	78.60 (02)	4.122	0.1706
Reproduction traits-						
Age at first conception (day)	609.45^b^ (11)	-	640.09^b^ (11)	407.00^a^ (01)	37.426	0.01243
Gestation length (day)	144.64 (11)	-	145.64 (11)	147.00 (01)	0.55	0.2038
Litter size (number)	1.30 (11)	-	1.27 (11)	1.00 (01)	0.133	0.7064
Age at first lambing (day)	752.27^b^ (11)	-	775.18^b^ (11)	554.00^a^ (01)	35.75	0.009183
Average lambing interval (day)	585.5 (08)	-	473.4 (05)	326.0 (01)	77.222	0.71893
Days open (day)	155.27 (11)	-	162.36 (11)	142.00 (01)	28.212	0.9924

BWT3 = Body weight at 3 months of age; BWT6 = Body weight at 6 months of age; BWT9 = Body weight at 9 months of age; BWT12 = Body weight at 12 months of age; ADG = Average daily weight gain; SEM = Standard error of the mean; Figure in parentheses indicates the number of observations. ^a,b^ means with different superscripts differed significantly within the row (*p* < 0.05).

**Figure 2.**
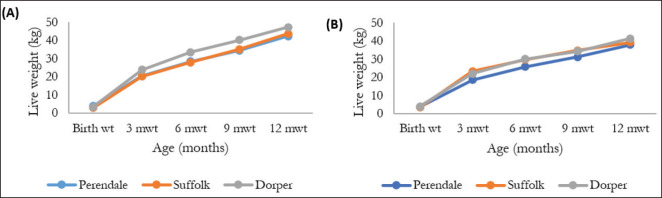
Cumulative growth performance of different exotic males (A) and females (B).

The cumulative growth performance of different exotic male and female sheep is shown in Figure 2. Among the males, the growth performance was highest in the Dorper breed except for the birth weight, which was found to be higher in the Perendale breed. The cumulative birth weight (kg) was 3.96, 2.88, and 3.42, and BWT12 (kg) was 42.32, 43.65, and 47.26 for the males of the Perendale, Suffolk, and Dorper breeds, respectively. Although the Dorper female was superior in growth performance among the breed, the Suffolk was also found to be superior in the cases of BWT3 and BWT9. The cumulative birth weight (kg) was 3.59, 3.42, and 4.03, and BWT12 (kg) was 37.98, 39.3, and 41.3 for the females of the Perendale, Suffolk, and Dorper breeds, respectively. In both sexes, the highest growth performance was observed in the Dorper breed, followed by the Suffolk and Perendale breeds, respectively. The average birth weight of females was higher than that of males, but the overall growth performance was found to be higher in males. Compared to the current result, higher birth weight (4.26 kg) and BWT3 (22.65 kg) were found, but in the case of BWT12, a lower value was reported for Perendale sheep [22]. In another study [23], the average BWT3 of Perendale sheep was found to be 31.33 kg, which is much higher than the current findings. In the case of Suffolk sheep, the average birth weight (BWT3) and average daily gain (ADG0-3) of the present study were significantly lower than the findings reported in [24], which listed the birth weight (BWT3) and average daily gain (ADG0-3) as 4.94 kg and 300 gm/day, respectively. In other studies [25,26], birth weights of 5.7 and 4.8 kg were found for Suffolk sheep, which are also significantly higher than the current findings. Relatively higher BWT6 was found (48.7 kg) compared to the present findings [27].

However, a relatively lower birth weight (3.33 kg) of Dorper sheep was reported [19], whereas higher birth weights were also reported [25,28,29] with values of 4.12, 5.5, and 4.09 kg, respectively, compared to the current findings. Likewise, a similar BWT3 (23.8 kg) but higher ADG0-3 (219.0 gm/day) than the present study was reported [28]. The average BWT6, BWT9, and BWT12 of Dorper sheep were noted as 36.7, 47.8, and 63.5 kg, respectively [29], which is much higher than the present result. Likewise, the wool production of Perendale sheep was significantly lower than the findings [22] that reported an average wool production of 2.75 kg per sheep. The variations in the results may be attributed to several factors, including geographical differences, environment, management, and adaptation. In general, the performance of any breed decreases compared to its country of origin when it is transported to another country. As the population size was very small, especially unbalanced among the breeds, more research is needed for a better understanding.

### Reproductive performance

The reproductive performance of sheep may vary due to several factors, including the breeding potential of the breeding season, nutritional state, environment, location, health status, and other factors [28,30-32]. Table 2 shows the reproductive performance of different exotic sheep. There was no significant difference between breeds except for the average lambing interval. The lowest lambing interval was observed in Dorper sheep, followed by Suffolk and Perendale, with average values of 382.5, 383, and 635 days, respectively. The effect of season on different reproductive traits is narrated in Table 3. A significant effect of season was found in the cases of age at first conception and age at first lambing. The lowest age at first conception (day) and age at first lambing (day) were observed in the rainy season, followed by early summer and winter, and the average values were 407, 609.45, and 640.09; and 554, 752.27, and 775.18, respectively. Different research also found a significant influence of season on age at the first lambing [28,33]. A relatively lower age at the first lambing (346 days) was found by researchers [28], compared to the current findings. In another study [30], a higher age at the first lambing (day) was noted compared to the present result, as observed in short rain (676), long rain (653), and dry season (990). The current findings showed that the gestation length (days) of Suffolk (144.25) and Dorper (146.67) sheep was lower than the findings of another research [24,34], which reported 146.20 days for Suffolk and 147 days for Dorper, respectively. In the present study, the average litter size of Perendale sheep was found to be 1.25, which is lower than the findings [22] that reported an average litter size of 1.54. Likewise, in Suffolk sheep, a lower litter size (1.42) was found, which is lower than the findings reported as 1.63 and 1.8, respectively [24,35]. In the case of Dorper sheep, a lower litter size (1.22) was also found than the findings [34] that reported an average litter size of 1.54. The average lambing interval (in days) of Dorper sheep was found to be 382.5, which is significantly lower than the findings [30] that reported an average lambing interval of 398 days. Variation in lambing interval results in the significant effect of area, genotype, lambing season, parity, and the combined effect of different genetic and non-genetic factors [31]. However, breed-specific references for all reproductive traits were not found to compare with the current result. 

**Figure 3.**
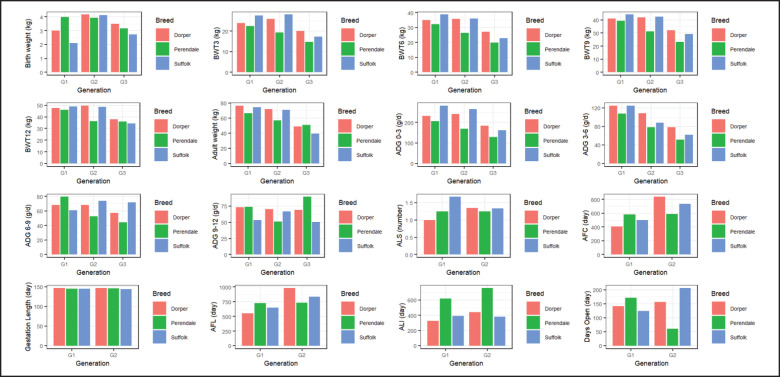
Generation-wise productive and reproductive performance of exotic sheep. (BWT3 = Body weight at 3 months of age; BWT6 = Body weight at 6 months of age; BWT9 = Body weight at 9 months of age; BWT12 = Body weight at 12 months of age; ADG = Average daily weight gain; ALS = Average litter size; AFC = Age at first conception; AFL = Age at first lambing; ALI = Average lambing interval.

Generation-wise productive and reproductive performance of different exotic sheep is described in Figure 3. Since the number of observations varied, no consistent trend was observed in different traits across successive generations. The performances did not reveal any significant trend that would facilitate a concrete selection decision. In general, it took a comparatively longer duration to produce two successive generations in our country context. However, no such review was found that describes the performance of the studied sheep breed by generation.

### Disease and survivability of exotic sheep

The most frequently occurring disease was fungal infection (42.37%), followed by lameness (16.94%), mastitis (13.56%), nutrient deficiency (10.17%), infection in male genital organs (6.78%), fever (3.39%), dysentery (1.69%), bloat (1.69%), tumor (1.69%), and myiasis (1.69%). The occurrence of fungal infections might be attributed to the country's hot and humid climatic conditions. Within the breed, the highest disease frequency was observed in Perendale sheep, followed by Dorper and Suffolk sheep. No such review was found that describes the disease frequency of the aforementioned sheep breed.

**Figure 4.**
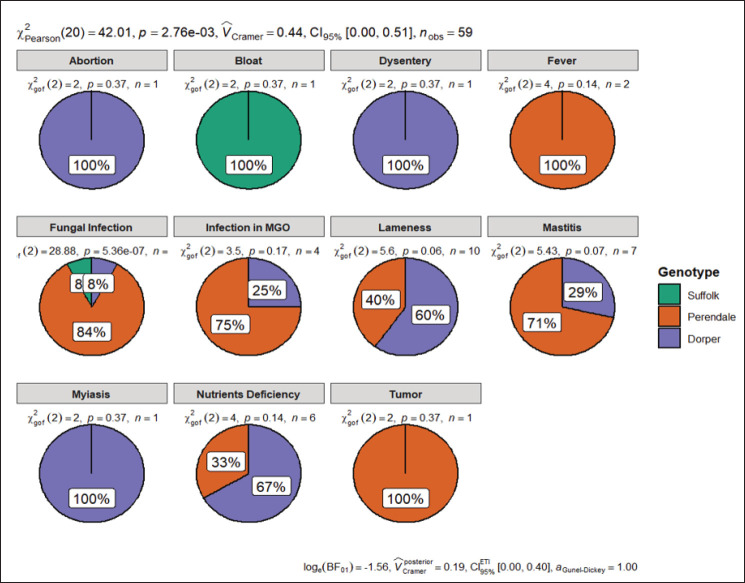
Disease frequency of different exotic sheep. (*MGO= Male genital organ).

Table 4 shows the survivability of different exotic sheep. Among all the breeds, survivability was higher in the growing stage compared to the lamb stage. The average survivability rate of lambs and growing sheep was 96.34 and 98.73, respectively. The survivability of Dorper sheep was found to be significantly higher than the previous findings [25,28,34], which were recorded as 81%, 91%, and 93.4%, respectively. Although no previous review was found for Perendale and Suffolk sheep, the survivability rate was also found to be appreciable for these breed (Fig. 4).

**Table 4.** Survivability (%) of exotic sheep.

Breed	Lamb (0–3 months of age)		Growing (3–8 months of age)	
	Survivability (%)	Mortality (%)	Survivability (%)	Mortality (%)
Perendale	96.3 (52)	3.70 (02)	98.08 (51)	1.92 (01)
Suffolk	92.31 (12)	7.69 (01)	100 (12)	0
Dorper	100 (15)	0	100 (15)	0

## Conclusion

The performance of exotic sheep varies not only due to their genetic makeup but also to non-genetic factors such as environment, management, and others. Although the productive and reproductive performances of the exotic sheep are lower than in their country of origin, they are well adapted to our country's environment. Furthermore, the maximum population size of Perendale sheep in successive generations indicates a high degree of adaptation of the breed compared to Dorper and Suffolk. The highest growth performance of Dorper sheep suggests the breed could be useful for producing meat-type crossbred sheep. As the sample size is small, no concrete decision could be made regarding selection. By applying the genetic and non-genetic improvement model, superior exotic sheep can be incorporated to enhance the native sheep of Bangladesh.

## List of abbreviations

ADG, average daily weight gain; ADG0-3, average daily weight gain at 0-3 months of age; ADG3-6, average daily weight gain at 3-6 months of age; ADG6-9, average daily weight gain at 6-9 months of age; ADG9-12, average daily weight gain at 9-12 months of age; BWT3, body weight at 3 months of age; BWT6, body weight at 6 months of age; BWT9, body weight at 9 months of age; BWT12, body weight at 12 months of age; %, percentage; gm, gram; gm/day, grams per day; Kg, kilograms; SEM, Standard error of the mean.
